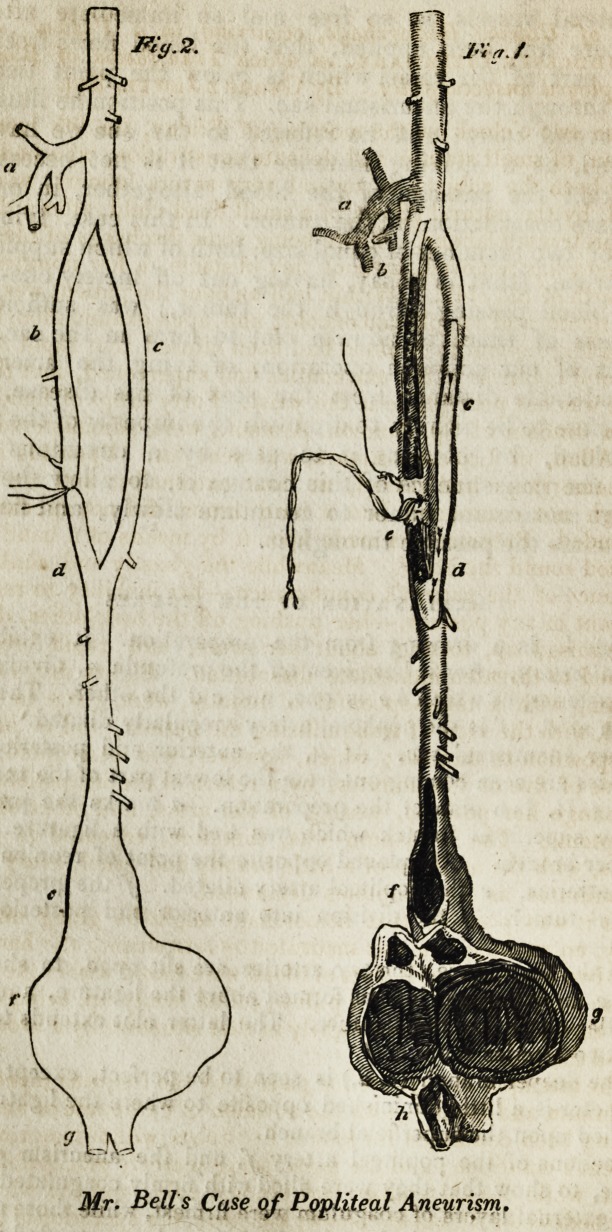# A Case of Popliteal Aneurism, in Which the Femoral Artery Was Found to Be Divided into Two Trunks, Which Again Became Reunited Where the Vessel Passes through the Tendon of the Triceps Muscle

**Published:** 1826-08

**Authors:** C. Bell

**Affiliations:** the Middlesex Hospital


### ANEURISM.

*A Case of Popliteal Aneurism, in which the Femoral Artery was found to be divided into two Trunks, which again became re-united where the Vessel passes through the Tendon of the Triceps Muscle.* Treated at the MIDDLESEX HOSPITAL, by Mr. C. BELL.

FEBRUARY 18th, 1826.— —— Adams, a large and muscular negro, was admitted into the Middlesex Hospital, having a pulsating tumor situated at the upper part of the calf of his left leg, just below the knee-joint. He did not know his age, but he seemed to be between forty and forty-five. He first perceived the tumor four years ago; it was then small, and not attended with pain or inconvenience. About a month ago the swelling suddenly became larger, and he experienced great numbness in the leg and foot.

The situation of the tumor is unusual, being not properly in the popliteal cavity, but further down. It is more superficial than a common popliteal aneurism, and rises out between the heads of the gastrocnemius.* The artery at the groin is very large, and easily felt. On compressing it, the pulsation of the tumor can be stopped; but the pulsation cannot be stopped by pressing on the middle of the thigh. For these reasons, Mr. Bell stated that he should tie the artery lower in the thigh than usual.

*Operation.*—On Monday, 20th February, the operation was performed. It was attended with no unusual circumstances. The artery was easily found by lifting the edge of the sartorius muscle, and neither the vein nor nerve were exposed. After the ligature was applied, the pulsation of the artery against it was distinctly observed by all who were near the patient. As an arterial branch arose from the trunk close above the ligature, it was purposely cut across. It threw out its blood with great force, and was secured.†

The moment after the artery was tied, Mr. Shaw, who had his hand on the tumor, said that the pulsation was stopped; and, on asking the patient what he felt, he immediately answered, "There is no more painful beating." But Mr. Shaw, keeping his hand on the tumor, felt the pulsation distinctly return in a few seconds: and so distinct was the pulsation, that he remarked it to Mr. Bell; who, after putting his hand upon the tumor, and observing that the ligature was moved by the regular pulsation of the artery, replied, "Well, be it what it may, I shall do no more: we have done all we ought to do." The edges of the wound were brought together with short adhesive straps, and the patient was carried to bed. On examining the tumor half an hour afterwards, the pulsation was nearly as distinct as before the operation, and quite different from that *thrill*, or slight pulsation, which is so frequently found after this operation.

23d.—The pulsation of the tumor stopped this morning, (the third after the operation.) Hitherto the patient had not suffered in consequence of the operation: but he is now ill and feverish; he has a cough, and complains of pains through the body; the wound is not uniting kindly. A very remarkable impression seems to have been made on his constitution, and this at the very time of the pulsation of the tumor stopping.

* In the figure it may be seen that the popliteal artery is enlarged above the proper aneurism. The point where it began to be dilated was opposite to the usual place for popliteal aneurism to form. This was ascertained on removing the parts from the body.

† Mr. Bell, in his clinical report on this subject, stated that, when we could discern a branch coming off in this manner, we ought either to apply. the ligature upon the main artery above the branch, or to take up the branch separately; for if the blood be permitted to take its course through that branch, the coagulum is prevented from forming to any extent, and therefore the ligature upon the main artery becomes insecure.

24th.—He is very ill. The short cough, which he has had for some time, is much worse. He has pain; but whether in his stomach or chest, cannot be exactly determined, from the indistinctness of his account of it.—Six ounces of blood have been taken from his arm ; and he is ordered to have an opiate linctus, to relieve his cough.

26th.—Gradually sunk, and died this morning. The whole line of the sartorius muscle had become swollen and tender, and a serous effusion distilled from the wound.

During the progress of this case, no thermometrical observation was made on the heat of the limb; which throughout felt hotter than the other. It was obvious that the circulation through it was completely restored.

*Dissection.*—The sartorius muscle, from end to end, was affected with inflammation, of an erysipelatous character, which had spread along the whole course of its sheath. The muscle itself was swollen, and tumid with serous effusion.

Just below the part where the profunda was given off, the femoral artery was divided into two nearly equal branches. These ran down parallel to each other, to the part where the artery passes through the tendon of the triceps muscle: here they reunited. The ligature was found on the more superficial artery, a little above this reunion.

At his next clinical lecture, Mr. Bell made the following remarks : There was no delay nor difficulty during the operation; the sartorius was lifted up, the fascia covering the artery opened, and the sheath of the artery dissected, by scratching with the point of the knife, and carrying the back of the knife forward. If this had not been done with precision, we see that very awkward circumstances might have occurred by cutting the deeper artery at the point of reunion.

The surprise is now, not that the tumor should have continued to beat, but how it should have ceased to beat by a ligature being put only on one of the arteries, either of which was fully sufficient for the circulation of the limb.

It was Mr. Bell's opinion that the unfavourable termination in this case arose not from the constitution sympathising with the condition of the tumor, but in consequence of the very peculiar condition of the sartorius muscle, and the fever which it produced ; and that to the weakness of the circulation thence arising, the coagulation of the blood in the aneurism, and consequent stopping of its pulsation, were to be attributed.

This case was brought forward by Mr. Bell in his lecture before the College of Surgeons, on Tuesday, 9th May, in illustration of the pathology of aneurism. He stated that, in the operation, the effect of a ligature applied on the main artery is not, as is generally supposed, to prevent the blood from flowing through the sac. For the circulation through the collateral vessels is so free and so immediate after the ligature has been applied, that the blood flows freely into that part of the vessel which is below the point tied, and also through the aneurismal sac. This position he illustrated by several cases; and he referred to the one we have just related, as a striking instance that it is not necessary to obstruct the passage of the blood altogether, in order to procure coagulation in the tumor. In this case, tying only one of two branches of equal size, both of which supplied the aneurism, (that is to say, having cut off merely one-half of the blood passing through the tumor,) was sufficient, in process of time, to allow a clot to form in the sac. The effect of our common operation, of tying the artery at a considerable distance from the seat of the disease, seems therefore to be merely to diminish the impetus of the stream of blood, by obliging it to pass by a circuitous route; and the consequence of this change is, to allow the blood in the aneurismal tumor to coagulate slowly, and finally to obliterate the passage through it.

EXPLANATION OF THE FIGURES.

Fig. I. Is a drawing from the preparation. It exhibits the crural artery, after it has given off the profunda *a*, dividing into two arteries, of which *b e* is one, and *c d* the other. They unite again at *d*. *f* is the popliteal artery irregularly dilated. *g* is the proper aneurismal sac. At *h*, the anterior and posterior tibial arteries are seen coming out from the lowest part of the tumor.

Fig. II. Is a plan of the preparation. *a* marks the profunda. *b* the superficial branch which was tied with a ligature. *c* the deeper branch. *d* is placed opposite the point of reunion of the two arteries. *e* the popliteal artery dilated. *f* the proper aneurismal tumor. *g* the division into anterior and posterior tibial arteries.

In the first figure, the two arteries are slit open, to show that in *b e* an extensive clot has formed above the ligature, and also a smaller clot below the ligature. The latter clot extends to where the two arteries reunite.

The deeper branch (*c*, *d*,) is seen to be perfect, except that its diameter is a little diminished opposite to where the ligature was applied upon the superficial branch.

Sections of the popliteal artery *f*, and the aneurism *g*, were made, to show that they were filled with firmly coagulated blood. The external layers of coagulum were firmest, while those near the centre were comparatively soft. The coagulum extended into the tibial arteries to the extent of about an inch below the tumor.

*Mr. Bell's Case of Popliteal Aneurism.*

## Figures and Tables

**Fig. 2. Fig. 1. f1:**